# Comparison of InterTAN and PFNA internal fixation for elderly patients with intertrochanteric fracture: A retrospective cohort study

**DOI:** 10.12669/pjms.40.4.8794

**Published:** 2024

**Authors:** Li Li, Xing Fang, Jie Gao, Weizhen Han, Xiangan Kong

**Affiliations:** 1 Li Li, Department of Orthopedics, The Second People’s Hospital of Hefei, Hefei Hospital Affiliated to Anhui Medical University, 246 Heping Road, Hefei City, Anhui Province 230011, P.R. China; 2 Xing Fang, Department of Orthopedics, The Second People’s Hospital of Hefei, Hefei Hospital Affiliated to Anhui Medical University, 246 Heping Road, Hefei City, Anhui Province 230011, P.R. China; 3 Jie Gao, Department of Orthopedics, The Second People’s Hospital of Hefei, Hefei Hospital Affiliated to Anhui Medical University, 246 Heping Road, Hefei City, Anhui Province 230011, P.R. China; 4 Weizhen Han, Department of Orthopedics, The Second People’s Hospital of Hefei, Hefei Hospital Affiliated to Anhui Medical University, 246 Heping Road, Hefei City, Anhui Province 230011, P.R. China; 5 Xiangan Kong, Department of Orthopedics, The Second People’s Hospital of Hefei, Hefei Hospital Affiliated to Anhui Medical University, 246 Heping Road, Hefei City, Anhui Province 230011, P.R. China

**Keywords:** InterTAN, Proximal femoral nail antirotation, Internal fixation, Intertrochanteric fracture, The elderly

## Abstract

**Objective:**

To compare the clinical outcomes of InterTAN nail and proximal femoral nail antirotation (PFNA) internal fixation for the treatment of intertrochanteric fractures in the elderly.

**Methods:**

We retrospectively reviewed the clinical records of 151 elderly patients with intertrochanteric fractures treated in The Second People’s Hospital of Hefei from October 2019 to December 2021. Among them, 73 patients had undergone InterTAN (InterTAN group) and 78 patients had undergone PFNA (PFNA group) internal fixation. Operation-related variables (operation time, incision length, intraoperative bleeding volume, hospital stays length, and fracture healing time), complications, and Harris scores were compared between the two groups.

**Results:**

The operation time and incision length were shorter and the intraoperative bleeding was less in the PFNA group than in the InterTAN group (all *P-values* <0.05), but the fracture healing time was longer in the PFNA group (*P*<0.05). We found similar hospital stays and surgical complications in the two groups (*P*>0.05). In addition, the Harris hip joint scores were significantly higher in the InterTAN group than in the PFNA group at one, six, and twelve months after the operation (*P*<0.05).

**Conclusions:**

InterTAN and PFNA internal fixation have their own advantages in treating patients with intertrochanteric fractures. InterTAN has better postoperative recovery results, while PFNA has less perioperative trauma. Clinically, InterTAN or PFNA should be selected based on the specific conditions of each patient to maximize the therapeutic benefit of each treatment method.

## INTRODUCTION

Intertrochanteric fractures are common in the elderly with high disability and mortality rates.^1^,^2^ Clinical studies have shown that the mortality within one year after an intertrochanteric fracture can reach 20% in the elderly, and approximately 40% of the surviving patients are left with reduced ability to move independently.^3^,^4^ Therefore, the goal of the treatment should be to reduce the mortality and restore the function of the hip joint as soon as possible to promote independent mobility.^5^

Surgical operations are the preferred treatment for treating patients with intertrochanteric fracture, and intramedullary fixation is a common procedure.^6^ Its biomechanical advantages and avoidance of periosteum peeling can effectively promote fracture healing.^5^,^6^ InterTAN and PFNA are commonly used intramedullary fixation techniques.^7^ InterTAN head and neck screws feature a high anti-rotation ability due to their interlocking combination design.^6^,^7^ The spiral blade of PFNA provides strong anchoring force and a good compression effect on the fracture, thereby enhancing biomechanical stability.^7^,^8^ However, recent meta-analysis by Wang et al^9^ and Lui et al^10^ has showed conflicting results on the pros and cons of these two devices. Therefore, in this manuscript we analyzed the outcomes of elderly patients with intertrochanteric fracture who received surgical treatment in our hospital from October 2019 to December 2021.

## METHODS

We retrospectively selected the records of 151 elderly patients (66 men and 85 women) with intertrochanteric fracture treated in The Second People’s Hospital of Hefei from October 2019 to December 2021 for review. The average age of the patients was 68.26±5.05 years; 73 received InterTAN internal fixation, (InterTan group) and 78 cases received PFNA internal fixation (PFNA group).

### Inclusion criteria:

- Digital and conventional X-ray imaging examinations confirmed the diagnosis of intertrochanteric fracture (AO/ATO type 31A2-A3), and all of the patients had an acute intertrochanteric fracture^11^
- Age ≥ 60 years
- Patients with a minimum of one year follow-up
- Individual able to move freely before the fracture event
- Absence of other combined injuries

### Exclusion criteria:

- Patients with pathological fractures
- Patients with injury of femur or hip joint
- Patients with hip arthritis, lumbar disc herniation, or other diseases affecting hip joint function
- Patients with malignant tumors
- Patients with American Society of Anesthesiologists (ASA) score of IV or V.
- Patients missing medical records and follow-up data.

### Ethical Approval

The Medical Ethics Committee of Hefei Second People’s Hospital approved this study (2023-Science-019, date: 2023-03-15).

All patients were treated with intramedullary fixation surgery by the same group of surgeons. After anesthesia induction, the patients were positioned supine and underwent closed reduction of the pelvis with a fixed assistant. The operator then flexed the knee and hip to 90°, held the popliteal fossa, and applied upward traction to correct the shortening deformity. Hip extension, internal rotation, and abduction were performed to correct the angular deformity. After obtaining fluoroscopy confirmation of the reduction, the affected limb was fixed on a traction bed, and the operation area was routinely disinfected and covered with towels.

### InterTAN (Smith & Nephew, Memphis, Tennessee)

**Fig.1:**
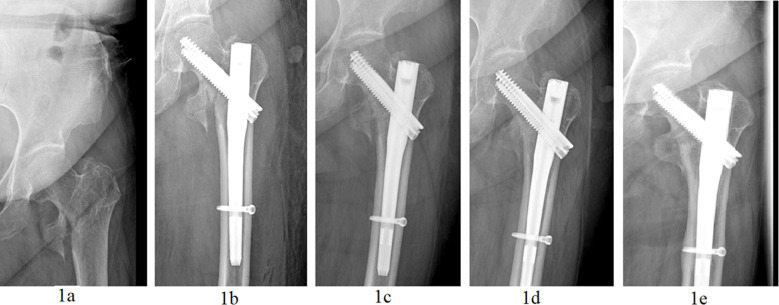
InterTAN internal fixation in a 71-year-old female patient with a left intertrochanteric fracture. 1a: X-ray image showing intertrochanteric fracture of the femur after the injury. 1b: X-ray image immediately after the operation showing appropriate fracture reduction. 1c: X-ray image showing appropriate internal fixation evolution one month after the operation. 1d: X-ray image showing bone union at the fracture site six months after the operation. 1e: X-ray image showing appropriate fracture healing 12 months after the operation.

The surgeon performed a 5cm longitudinal incision at the lateral side of the apex of the greater trochanter of the femur, and exposed the greater trochanter. After selecting the insertion point where the apex of the greater trochanter deviates slightly to the inside, the surgeon used an opener to expand the bone opening window and inserted the guide needle into the medullary cavity. After that, the surgeon carried out a selective reaming treatment (according to each specific situation) and inserted the main intramedullary nail. Next, the surgeon percutaneously inserted the other guide needle in the direction of the femoral neck. After confirming satisfactory results with the C-arm of the X-ray fluoroscopy machine, a guide needle and a lag screw were inserted in the direction of the two guide pins. The guide needle was removed, and the proximal end of the femur was locked for compression fixation. After achieving the ideal effects according to the fluoroscopy images, the surgeon inserted a lag screw at the distal end of the intramedullary nail. Once the fluoroscopy images had shown its position behind the locking hole, the surgeon screwed in the nail cap of the main nail. After fluoroscopy confirmation of the fracture reduction and satisfactory internal fixation, the area was suture closed and the incision bound to complete the operation, Fig.1

### PFNA (Synthes, Solothurn, Switzerland)

**Fig.2:**
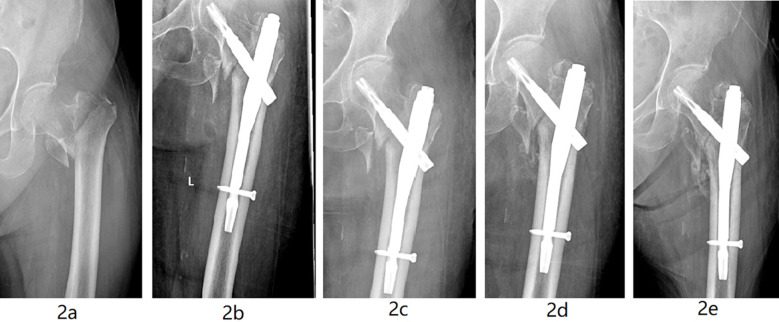
PFNA internal fixation in a 68-year-old female patient with a left intertrochanteric fracture. 2a: X-ray image showing intertrochanteric fracture of the femur after the injury. 2b: Postoperative X-ray showing appropriate fracture reduction. 2c: X-ray image showing appropriate internal fixation position evolution one month after the operation. 2d: X-ray image showing appropriate bone union of fracture six months after the operation. 2e: X-ray image showing appropriate fracture healing 12 months after the operation.

The surgeon performed a 5cm longitudinal incision on the outside of the apex of the greater trochanter of the femur, dissected the tissues to expose the greater trochanter, and selected an insertion point slightly medial to the apex of the greater trochanter. After performing bone opening window treatment, the surgeon inserted a guide needle into the medullary cavity, confirmed the insertion point (behind the medullary cavity) by fluoroscopy, and conducted selective reaming treatment. The intramedullary nail was then inserted under the guidance of the guide pin, followed by a second guide pin. The spiral blade was inserted towards the guide pin, which was positioned in the middle of the femoral head and tibia (as confirmed by fluoroscopy) and locked. Next, the surgeon inserted a lag screw into the distal end of the intramedullary nail located in the locking hole, and screwed the main nail cap. Fracture reduction and internal fixation were confirmed by fluoroscopy, and the incision was washed, sutured, and bound, to complete the operation, Fig.2.

### Postoperative care

The postoperative care was same in both groups. All patients received prophylactic anti-infective treatment for 24 hours after surgery. Continuous passive motion was applied twice daily by a physiotherapist after surgery. Patients were encouraged to engage in out-of-bed activities and partial weight-bearing as recommended by the surgeons on the following days.

We collected basic clinical data on the intraoperative and postoperative condition of patients including 1) intraoperative operation time, incision length, intraoperative bleeding, hospital stay length, and fracture healing time; 2) postoperative complications: coxa vara, incision infection, delayed union of fracture, deep vein thrombosis, and loose and broken internal parts; and (3) postoperative rehabilitation according to the Harris hip joint score^12^ (used to evaluate the postoperative rehabilitation with a maximum total score of 100 points for the best hip joint function).

### Data Analysis

We used SPSS v26.0 (SPSS, Chicago, IL, USA) for all statistical analyses. We compared categorical variables via Fisher’s exact or Chi-squared tests. Continuous variables with normal distribution were expressed as means ± standard deviation (SD) and were compared via Student’s *t* test, while continuous variables with non-normal distribution were expressed as median (interquartile range) and Mann-Whitney’s *U* tests. We considered *p* <0 .05 as the significance threshold.

## RESULTS

We analyzed the records of 151 patients: The InterTAN group included 34 men and 39 women aged 60 to 81 years (average 67.56±4.64 years), the injury site was the right one in 46 cases and the left side in 27 cases. The causes of injury included falls (63.0% or 46 of 73), traffic accidents (26.0% or 19 of 73), falls from heights (8.2% or 6 of 73), and others (2.7% or 2 of 73). The PFNA group included 32 men and 46 women aged 60 to 82 years (average 68.91±5.34 years), the injury site was the right one in 36 cases and the left one in 42 cases. The causes of injury included falls (74.4% or 58 of 78), traffic accidents (19.2% or 15 of 78), falls from heights (3.8% or 3 of 78), and others (2.6% or 2 of 78). We found similar values for the general data variables between the two groups (*P*>0.05) (Table-I). The mean operation time and incision length in the PFNA group were shorter than in the InterTAN group, and the mean intraoperative bleeding was less than that in the InterTAN group (*P*<0.05). However, the mean fracture healing time in the PFNA group was longer than in the InterTAN group (*P*<0.05), we found similar hospital stay lengths in the two groups (*P*>0.05) (Table-II). The incidence of complications in the InterTAN group was 4.11%, a value similar to that of the in PFNA group at 5.13% (*P*>0.05; Table-III). At one, six and twelve months after the operation, the mean Harris hip joint scores of the two groups were significantly higher than those before the operation, with those of the InterTAN group being significantly higher than those of the PFNA group (*P*<0.05) (Table-IV).

**Table-I:** Comparison of general data between the two groups.

Group	n	Gender (Male/ Female)	Age (Years)	Fractured side		Cause of injury			

				Right	Left	Fall	Traffic accident	Falling from height	Others
InterTAN group	73	34/39	68.00(64.00, 70.00)	46	27	46	19	6	2
PFNA group	78	32/46	68.50(65.00, 72.00)	36	42	58	15	3	2
*χ^2^*/*Z*		0.472	-1.624	1.303		2.693			
*P*		0.492	0.104	0.254		0.441			

**Table-II:** Comparison of operation-related variables between the two groups.

Group	Operation time (minute)	Incision length (cm)	Intraoperative bleeding volume (mL)	Hospital stays length (days)	Fracture healing time (weeks)
InterTAN group(n=73)	50.08±7.14	7.35±1.22	164.00 (158.50,171.00)	13.00 (10.50, 15.00)	12.00(11.00, 13.00)
PFNA group(n=78)	40.82±6.66	6.28±1.12	125.00(119.00, 130.00)	13.00(11.00, 15.00)	13.50(12.00, 15.00)
*t/Z*	8.245	5.667	-10.586	-0.975	-3.754
*P*	<0.001	<0.001	<0.001	0.329	<0.001

**Table-III:** Comparison of operation complications between the two groups [*n* (%)].

Complication							

Group	n	Coxa vara	Incision infection	Delayed union of fracture	Deep vein thrombosis	Loose and broken internal parts	Total
InterTAN group	73	1 (1.4)	1 (1.4)	1 (1.4)	0 (0.0)	0 (0.0)	3 (4.2)
PFNA group	78	1 (1.3)	1 (1.3)	1 (1.3)	0 (0.0)	1 (1.3)	4 (5.2)
*χ^2^*	-	-	-	-	-	-	0.086
*P*	-	-	-	-	-	-	0.766

**Table-IV:** Comparison of Harris scores between the two groups before and after the operation.

Group (n)	Preoperative	One month after operation	Six months after operation	Twelve months after operation
InterTAN group (n=73)	45.00(42.00,49.00)	76.72±5.73^a^	78.45±6.12^a^	85.56±5.68^a^
PFNA group (n=78)	46.00(42.75,51.00)	67.52±5.88^a^	74.68±6.52^a^	79.86±5.49^a^
*t/Z*	-0.785	9.730	3.660	6.276
*P*	0.432	<0.001	<0.001	<0.001

***Note:*** Compared with the pre-operative value within each group,

a P<0.05.

## DISCUSSION

We found shorter operation times and incisions, and less intraoperative bleeding in the PFNA group, while the fracture healing times were shorter and the hip joint recovery after operation were better in the InterTAN group.

Yu C et al.^7^ included 12 studies in a meta-analysis, comparing outcomes in 1015 patients after InterTAN and PFNA; they found shorter operation times and less intraoperative bleeding in the PFNA group, but the total complications and internal fixation failures were less after InterTAN, despite significant differences in the surgical incision lengths, fracture healing times, and other postoperative complications. However, their Harris scores at six and 12 months after the operations were similar in both groups. Conversely, a most recent meta-analysis by Wang et al.^9^ found that there is no significant difference between InterTan and PFNA and PFNA-II in terms of intraoperative blood loss, hospital stay, and postoperative Harris score. The conflicting results between these two studies and ours may be due to a) different study design: Yu’s study included randomized controlled trials (RCTs), Wang’s study included RCTs and non-RCTs, and ours was a single-center retrospective study; b) different sample sizes: as meta-analysis, Yu and Wang’s studies have compiled the study population together which is larger than ours.

The InterTAN system design is based on the handle of joint prostheses. The main screw has a trapezoidal cross section and a proximal end with a 4º valgus angle, consistent with a minimally invasive approach to the greater trochanter apex. This design provides biomechanical advantages and effective protection for normal fractures and surrounding soft tissue, promoting effective healing.^13^,^14^ The research of Albaker AB et al.^15^ shows that InterTAN fits the biomechanical structure of the human body well, and it can withstand cyclic loads that are 3.5 times the body weight. By contrast, PFNA was developed from PFN with the head and neck screws replaced by spiral blades, which can get directly attached to the femoral head without removing bone. The holding force is improved, and the distal screw can be dynamically locked, producing dynamic compression on the fracture end and promoting healing.^16^ In terms of hospital stay and complications, we found similar values in both groups, a difference from the study of Ma HH et al,^17^ in which patients receiving PFNA treatment had shorter hospital stays, and more complications than those undergoing InterTAN procedures. PFNA may produce less trauma than InterTAN, shortening hospital stays; but InterTAN shows good biomechanics and a stable internal fixation, which could enable patients to get out of bed soon reducing the complications caused by long-term bed rest. In addition, in terms of the functional recovery of the hip joint after the operation, we found significantly higher Harris hip joint scores in the two groups at 1, 6, and 12 months after the operation than before the procedure, and the inter-group comparison showed that the patients in the InterTAN group had significantly higher scores, a result similar to that of the study by Fukuoka N et al.^18^ suggesting that, while both InterTAN and PFNA internal fixation can promote fracture healing in elderly patients with intertrochanteric fracture and improve hip joint function, the InterTAN hip joint recovery effect is superior.

The treatment goal for elderly intertrochanteric fractures is to maximize the hip joint function, improve the patient’s ability to move independently, and reduce the disability and mortality rates.^19^ InterTAN can further enhance the rotational stability of the intramedullary nail, effectively fix the fracture, and promote fracture healing, enabling the patient to carry out effective functional exercise as soon as possible, and further improving the function of the hip joint.^20^ InterTAN and PFNA internal fixations have their own advantages in the treatment of elderly patients with intertrochanteric fractures. Although InterTAN necessitates long operation times and produces relatively large trauma, it provides appropriate hip joint function recovery outcomes after the operation, and its effects on the fixation strength, stability, and torque resistance are better than those of PFNA. By contrast, PFNA results in less trauma, and is more suitable for patients with poor physical conditions and low surgical tolerance.^21^,^22^

Our findings showed that InterTAN and PFNA internal fixation have their own advantages in treating patients with intertrochanteric fracture, which suggesting that when choosing InterTAN and PFNA internal fixation for elderly patients with intertrochanteric fracture, the more beneficial one of the two methods should be rationally selected according to the comprehensive physical examination results of the patients and the patients’ wishes. PFNA may be more appropriate for patients who are less tolerant of surgery, whereas InterTAN may be a better choice for patients who are in relatively good health and/or who require greater functional recovery of hip function.

### Limitations of this study

This was a single-center retrospective analysis. Our sample was small, and selection bias was unavoidable. In addition, residual confounding factors in the data collection process may have affected our results. Therefore, a high-quality, large-sample, prospective multi-center study is needed to verify the conclusions of this paper. Moreover, differences between functional outcome of the two devices with respect to age, side, fracture type and obesity etc could be further studied in future research.

## CONCLUSION

InterTAN and PFNA internal fixation have their own advantages in the treatment of patients with intertrochanteric fracture. InterTAN has better postoperative recovery results, while PFNA has less perioperative trauma. Clinically, InterTAN or PFNA should be selected based on the specific conditions of each patient to maximize the therapeutic benefit of each treatment method.

### Authors’ contributions:

**LL and XF** conceived and designed the study.

**JG, WH and XK** collected the data and performed the analysis.

**LL and XF** were involved in the writing of the manuscript and are responsible for the integrity of the study.

All authors have read and approved the final manuscript.
